# Understanding the Quorum-Sensing Bacterium Pantoea stewartii Strain M009 with Whole-Genome Sequencing Analysis

**DOI:** 10.1128/genomeA.01509-14

**Published:** 2015-01-29

**Authors:** Wen-Si Tan, Chien-Yi Chang, Wai-Fong Yin, Kok-Gan Chan

**Affiliations:** a Division of Genetics and Molecular Biology, Institute of Biological Sciences, Faculty of Science, University of Malaya, Kuala Lumpur, Malaysia; b Interdisciplinary Computing and Complex BioSystems (ICOS) Research Group, School of Computing Science, Newcastle University, Newcastle upon Tyne, United Kingdom; c Centre for Bacterial Cell Biology, Medical School, Newcastle University, Newcastle upon Tyne, United Kingdom

## Abstract

Pantoea stewartii is known to be the causative agent of Stewart’s wilt, which usually affects sweet corn (Zea mays) with the corn flea beetle as the transmission vector. In this work, we present the whole-genome sequence of Pantoea stewartii strain M009, isolated from a Malaysian tropical rainforest waterfall.

## GENOME ANNOUNCEMENT

Quorum sensing (QS) is a term coined to describe the ability of bacteria to communicate in order to form a unified response within a population (1). The communication occurs when small diffusible molecules in a given bacterial population synchronize and stimulate a series of gene expressions that could drive different responses, such as the production of virulence factors (2, 3). Since QS plays a vital role for bacteria, it is therefore important to study the freshwater-inhabiting bacteria that exhibit QS properties, because freshwater can serve as a reservoir for microorganisms (3, 4). Pantoea stewartii has long been known for causing Stewart’s wilt in sweet corn, and epidemics in the 1990s led to significant economic losses for the corn seed industry (5). P. stewartii causes infection in corn by utilizing the ability to communicate and enhance the expression of virulence genes. In this study, the P. stewartii strain M009 was isolated from a waterfall environment (3). Whole-genome sequencing was conducted in order to further understand the genetic makeup of the QS system.

The genomic DNA of strain M009 was extracted using a MasterPure DNA purification kit (Epicentre Inc., Madison, WI, USA), while the quality of extracted DNA was determined by NanoDrop spectrophotometer (Thermo Scientific, Waltham, MA, USA) and a Qubit version 2.0 fluorometer (Life Technologies, Carlsbad, CA, USA). The purified DNA was subjected to whole-genome shotgun sequencing on an Illumina MiSeq personal sequencer (Illumina Inc., CA, USA) which generated 4,831,705 paired-end reads, and a trimming of the sequences provided 1,065,952 quality reads. The trimmed reads were *de novo* assembled with CLC Genomic Workbench version 5.1 (CLC Bio, Denmark). A total of 56 contigs with an *N*_50_ size of approximately 223,175 were generated.

The draft genome of the strain M009 isolate contained 4,821,705 bases, with an average coverage of 44-fold and a G+C content of 53%. Gene prediction was then performed with the prokaryote gene prediction algorithm by using Prodigal version 2.60 (6). A total of 4,307 open reading frames were predicted, while the 4 rRNAs (2 copies of 5S rRNA and one copy each of 23S rRNA and 16S rRNA) and 70 tRNAs were predicted with RNAmmer (7) and tRNAscan SE version 1.21 (8), respectively. Subsequently, the strain M009 sequence was annotated with RAST (9).

From the annotation results, the luxI and luxR homologues of strain M009 were predicted to be located at contig 5, where the *luxR* gene was located upstream of the contig. The whole-genome sequence allows deeper understanding of the genetic makeup of P. stewartii to determine the link between QS ability with plant pathogenicity and production of virulence factors (10, 11).

### Nucleotide sequence accession numbers.

This whole-genome shotgun project has been deposited at DDBJ/EMBL/GenBank under the accession number JRWI00000000. The version described in this paper is the first version, JRWI01000000.
